# Case of Pseudo-Syphilis

**Published:** 1807-03

**Authors:** 


### *To the Editors of the Medical and Phyfical Journal.*

GENTLEMEN,

A Few days after a suspicious connection, a Gentleman observed upon his prepuce, two small ulcers, with indurated edges and foul surfaces; the surrounding parts were much thickened, and the sores had the usual appearance of chancre. He was recommended to keep them clean, by frequent washing with soap and water, and to take small doses of calomel; the calomel in two or three days, was exchanged for the blue pill: but as neither form agreed with his bowels, and the sores were not at all amended

---

ed in the course of eight days, he was advised to leave off internal medicines, and rub in a drachm and a half of mercurial ointment daily.—From this time the ulcers progressively healed, and in a week were well: but he complained of uneasiness in his throat, when he swallowed; and upon looking into it, I observed the right tonsil highly inflamed, with an ulcer of a venereal aspect in the centre; the sore was deep, and a thick white matter adhered to its surface. The patient had been affected two or three times in the course of his life, both with gonorrhœa and chancre, but the inguinal glands had never suffered from absorption. He had been inattentive when unwell, and was disposed to consider this attack in his throat, as the secondary effect of a former infection: he had long, indeed, suspected that a venereal taint was lurking in his constitution, and was desirous to get rid of it, by undergoing a proper mercurial course. He now confined himself wholly to his room, and used a drachm and a half of mercurial ointment, morning and evening, for a fortnight: his mouth was slightly affected, and although the ulcer began to heal, another of a similar character made its appearance higher up on the same tonsil; the first soon got well, but the second spread till it was nearly the size of a sixpence, and then was stationary for about three weeks; by which time the salivary glands were highly excited, the sore shewed a disposition to heal, and in a few days was well. He now thought himself free from disease, yet, to prevent a relapse, continued the use of the ointment three or four evenings after the ulcer had disappeared; he then was attacked with an uneasy sensation in the left side of his throat: upon inspection, I found that tonsil inflamed, and when his head was placed in the position most convenient for an accurate examination, a large deep ulcer was discovered behind the tonsil, extending as far laterally as the eye could reach. This sore had as perfect a syphilitic appearance as the former.

The patient's mind became greatly depressed, he fancied he was completely poxed, had no faith in any remedy but mercury, and as that medicine had failed after so long a trial, he almost despaired of recovery. Wearied out as he was, by the length of his confinement, and debilitated by the violence of the mercurial action, I did not hesitate to recommend a change of plan. I have mentioned that he attempted to take calomel and the blue pill; he also tried corrosive sublimate, and a decoction of sarsaparilla, but they they all disagreed with him, and he abandoned them. He was now directed to use the warm bath once, for the purpose of cleaning himself from the filth of the inunction, to have recourse to exercise, adopt a more generous diet, and take as much nitric acid as he could bear. He began with two drachms daily, diluted in a sufficient quantity of water with sugar, to render it palatable. The acid agreed wonderfully well with him: I never beheld such rapidly beneficial effects from any medicine whatever, as were produced in this instance: in two days the appetite was improved, the general health amended, the inflammation on the tonsil abated, and the ulcer diminished, and presenting a more favourable aspect. In a week the sore was well; and in three weeks his health was sufficiently re-established, to allow him to discontinue the acid. More than two years have elapsed since this case occurred, and the subject of it is free from disease.

At the time this patient was under my care, I had not the least doubt of the disease being venereal; but since I have perused the valuable and ingenious observations on diseases resembling syphilis, by Mr. Abernethy, my belief is staggered.

That ulcers arising on the penis from impure sexual intercourse, and succeeded by buboes, sore throats, coronæ veneris, and other symptoms of lues venerea, all which have disappeared without the exhibition of mercury, have been noticed by many authors, is certainly true, yet, I think, Mr. Abernethy is entitled to praise, for calling the attention of surgeons to these cases, in the pointed manner which he has done.

I have heard an opinion advanced, that if diseases so much alike each other in their appearances, that the most experienced eye cannot perceive a distinction, are to be considered and treated as different, danger will accrue from that irresolution and delay which the uncertain nature of the complaint will produce in the minds of practitioners; and that frequently, in venereal cases, the benefit which mercury can bestow, will be withheld, from an erroneous idea that the symptoms are not those of syphilis, and will in a short time subside, without submission to a disgusting and unpleasant course of medicine; yet, all hazard will be readily avoided by any careful surgeon, and the advantages that may attend Mr. Abernethy's distinction ought not to be set aside by trifling objections.

Disorders of the genitals always create particular interest est in the minds of patients, and are often productive of the utmost distress. When a person supposes himself affected with syphilis, and the symptoms have resisted repeated applications of mercury, he is apt to imagine that the complaint is too deeply rooted in his system to be eradicated; he sinks under the most gloomy apprehensions, and becomes truly wretched.

On such occasions, it should be deemed the duty of the medical attendant, to soothe the feelings of the afflicted; he may impart comfort by intimating that the disease is probably not venereal, and inspire hope and confidence by relating the fortunate termination of many similar cases. Medicine and a due attention to corresponding regimen, can yet effect all that is wished. Whatever possesses the means to invigorate, should be called in aid; and as general strength is gained, the symptoms, if they really had not a syphilitic origin, may gradually disappear.

In cases of pseudo-syphilis, particularly in scrophulous habits, the nitric acid is a valuable remedy; I have witnessed its beneficial influence, when every other tonic disagreed with the stomach and bowels. — I generally give with it a large quantity of sugar; some patients have taken three ounces in the daily dose: whether any good effects may be attributed to this combination, that are not due to the acid alone, I leave others to decide.

I will conclude this letter with another instance of its success after the failure of mercury.

An officer applied to me about a month ago, with an ulcer, resembling chancre, upon the frænum, which he perceived a few days after coition with a girl of the town. I prescribed the unguentum hydrargyri, and his mouth was soon affected. The ptyalism was kept up about a fortnight without any amendment in the ulcer; on the contrary, it retained its ill-looking surface, had extended upon the glans on each side of the frænum, and occupied a space also upon the prepuce. I desired him to leave off mercury, and take during the day, two drachms of nitric acid, mixed with water and sugar. Healthy granulations appeared in a week, and the sore is now so nearly well, that to-morrow he will proceed on duty to Ireland.

J. S. S.

*Coventry, December* 15, 1806.

